# Underestimated Rate of Status Epilepticus according to the Traditional Definition of Status Epilepticus

**DOI:** 10.1155/2015/801834

**Published:** 2015-06-21

**Authors:** Cheung-Ter Ong, Yi-Sin Wong, Sheng-Feng Sung, Chi-Shun Wu, Yung-Chu Hsu, Yu-Hsiang Su, Ling-Chien Hung

**Affiliations:** ^1^Department of Neurology, Chia-Yi Christian Hospital, Chiayi, Taiwan; ^2^Department of Nursing, Chung Jen Junior College of Nursing, Health Science and Management, Chiayi, Taiwan; ^3^Department of Family Medicine, Chia-Yi Christian Hospital, Chiayi, Taiwan

## Abstract

*Purpose*. Status epilepticus (SE) is an important neurological emergency. Early diagnosis could improve outcomes. Traditionally, SE is defined as seizures lasting at least 30 min or repeated seizures over 30 min without recovery of consciousness. Some specialists argued that the duration of seizures qualifying as SE should be shorter and the operational definition of SE was suggested. It is unclear whether physicians follow the operational definition. The objective of this study was to investigate whether the incidence of SE was underestimated and to investigate the underestimate rate. *Methods*. This retrospective study evaluates the difference in diagnosis of SE between operational definition and traditional definition of status epilepticus. Between July 1, 2012, and June 30, 2014, patients discharged with ICD-9 codes for epilepsy (345.X) in Chia-Yi Christian Hospital were included in the study. A seizure lasting at least 30 min or repeated seizures over 30 min without recovery of consciousness were considered SE according to the traditional definition of SE (TDSE). A seizure lasting between 5 and 30 min was considered SE according to the operational definition of SE (ODSE); it was defined as underestimated status epilepticus (UESE). *Results*. During a 2-year period, there were 256 episodes of seizures requiring hospital admission. Among the 256 episodes, 99 episodes lasted longer than 5 min, out of which 61 (61.6%) episodes persisted over 30 min (TDSE) and 38 (38.4%) episodes continued between 5 and 30 min (UESE). In the 38 episodes of seizure lasting 5 to 30 minutes, only one episode was previously discharged as SE (ICD-9-CM 345.3). *Conclusion*. We underestimated 37.4% of SE. Continuing education regarding the diagnosis and treatment of epilepsy is important for physicians.

## 1. Introduction

Status epilepticus (SE) is a medical emergency with significant morbidity and mortality. The traditional definition of SE (TDSE) includes a single clinical seizure lasting at least 30 min or repeated seizures over a period of more than 30 min without recovery of consciousness [1, 2]. The incidence of SE is considered to be from 9 to 41/100000 per year, with the TDSE being used in most of these studies [3–7].

When seizures continued for over 30 min in experimental animals, structural injury to the central nervous system was identified. Therefore, neurologists suggested that a shorter time of seizure is recognized as adequate for establishing the clinical diagnosis and initiating treatment for SE; thus, the operational definition of SE (ODSE) was suggested [8]. It was defined as a generalized, convulsive seizure persisting more than 5 min or two or more discrete seizures between which there is an incomplete recovery of consciousness. Early treatment results in better outcomes for SE patients [9]. Because physicians may follow different definitions, this could influence the diagnosis and treatment of SE. In wanting to investigate the trends regarding the incidence of SE, one would need to know how many patients were not diagnosed as SE in previous studies. This study aimed to estimate how many patients had seizures lasting longer than 5 min but were not diagnosed as cases of SE because the physician followed the traditional definition.

## 2. Methods

### 2.1. Data Source

In this retrospective study, the seizure information of patients was collected from Chia-Yi Christian Hospital between July 1, 2012, and June 30, 2014. Chia-Yi Christian Hospital is an acute care, 1000-bed, teaching hospital in the central part of Taiwan. All patients discharged with ICD-9 codes for epilepsy (345.X, all extensions and all positions) were enrolled to verify whether they were SE cases. The study was approved by the Institutional Review Board of the hospital.

### 2.2. Definition of SE

We considered any seizure lasting at least 30 min or repeated seizures over a period of more than 30 min without recovery of consciousness as TDSE. When the seizure lasted between 5 and 30 min, it was diagnosed as underestimated SE (UESE) based on the operational definition of SE.

### 2.3. Case Ascertainment

All the patients were admitted from the emergency department of the hospital. When a patient arrived at the emergency room in the midst of an attack, intravenous benzodiazepine or anticonvulsant was given immediately. If the seizure did not subside, another intravenous phenytoin or valproic acid was also administered. The family members of seizure patients were asked for a detailed description of the sequence of the seizure episode, including the onset time and the course of the seizure. In the chart of patient, all the information gathered in the emergency department was recorded, including drug usage, the onset time, and the course of the seizure. Seizure duration was determined from a review of the medical records and ambulance call sheets. The ambulance call sheet included the details about the time of request for help, the time of arrival of the ambulance, and the condition of the patient. We were particularly vigilant about any seizures lasting more than 5 min and also those lasting more than 30 min. When there was a doubt about the duration of the seizure, the shorter duration of the seizure episode was adopted.

The Glasgow outcome scale [10] (GOS) was used to evaluate the outcome of the patients with SE at discharge. When the patient was younger than 6 years, the patient was defined as a good recovery subject/case when he/she recovered to the baseline prior to the episode of the seizure (GOS 5). We classified the etiology and seizure type according to the international league against epilepsy (ILAE) guidelines and the revised terminology in the ILAE commission [11–13].

### 2.4. Statistics

Statistical significance between TDSE and UESE was analyzed using the Chi-square or Fisher's exact test for categorical variables, the *t*-test for continuous parameters, and one way ANNOVA for cost of treatment and length of stay. MedCalc for windows version 12.3 (MedCalc software, Ostend, Belgium) was used for data analyses.

## 3. Results

During the 2-year period covered by this study, 198 patients admitted to the hospital had 256 episodes of seizures. In the 198 patients, there were one episode of seizure in 161, two episodes in 25, three episodes in 7, four episodes in 3, five episodes in one, and seven episodes in one patient. In the 256 episodes of seizure, intravenous (IV) benzodiazepine was given for 43, phenytoin for 24, valproic acid for 8, levetiracetam for 3, benzodiazepine and phenytoin for 27, benzodiazepine and valproic acid for 13, benzodiazepine and levetiracetam for 6, and benzodiazepine combined with two anticonvulsants for 2 episodes individually. Among those episodes, 157 episodes occurred for less than 5 min and 99 episodes persisted for more than 5 min. Among the 99 episodes, there were 62 episodes with the discharge code 345.3, including 61 episodes of seizure continuing for 30 min or longer and 1 episode lasting between 5 and 30 min.

**Figure 1:**
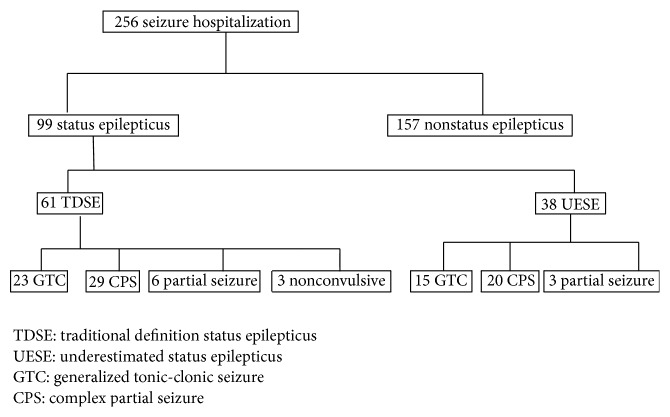
Flow chart of study inclusion.

According to the TDSE, 61 episodes of seizure were found in 56 patients. Thirty-eight episodes of seizure continued between 5 and 30 min in 34 patients, and they were defined as the UESE group (Table 1, Figure 1). Therefore, only 1 of 38 was previously diagnosed with SE (ICD-9-CM 345.3). The episodes of seizure occurred predominately in males and the complex partial seizure (CPS) was the major type defined by TDSE or UESE. Additionally, Table 2 shows that patients aged younger than 18 years were in a statistically significant manner categorized to belong to the UESE rather than TDSE group.

### 3.1. Seizure Duration

In patients with a first seizure lasting greater than 5 min, 33.3% (4/12) and 61.1% (11/18) of seizures lasted longer than 30 min in patients younger than 18 years and in patients older than 18 years, respectively. In contrast to the patients presenting with a first seizure, 52% (13/25) and 75% (11/44) of seizure episode lasted longer than 30 min in patients younger than 18 years and in patients older than 60 years with a history of epilepsy, respectively (Tables 3 and 4).

With respect to possible causes for the seizures, stroke and trauma histories were most commonly obtained. There were no significant differences between causes that led to SE being classified as TDSE or UESE (Table 5). The length of stay in the hospital was shorter in the UESE group (9.49 versus 3.71 days, *p* < 0.01), and the cost of medical treatment was significantly lower in the UESE group than in the TDSE group (55049 versus 18288 NT, *p* < 0.01) (Table 1).

## 4. Discussion

Data on the incidence, etiology, and mortality of SE are important for decisions involving allocation of institutional and even governmental resources, which may affect the strategy of primary and secondary prevention of SE. Although ODSE has been suggested for use in clinical practice, its effect on the incidence of SE is still unclear. In our hospital study, 62 episodes of seizure were discharged with a diagnosis of SE among 256 episodes of seizures, whereas 37 episodes were further defined as SE based on the ODSE.

Most studies investigating the incidence of SE were performed 10 years ago and followed the TDSE [3, 4, 6, 7]. Recently, two studies based on national data used the ODSE to investigate the incidence of SE [14, 15]. However, the two studies did not evaluate some patients who were not diagnosed with SE, even though their seizures lasted between 5 and 30 min. Therefore, it is important to investigate how many patients' seizures lasted more than 5 min but they were not diagnosed as having SE because the TDSE was applied. In this study, there were a total of 99 episodes of seizures continuing over 5 min, out of which 37 seizures were not compatible with the TDSE but were diagnosed with SE if the ODSE was followed. Our result implies that we may underestimate 37.37% (37/99) of episodes, although the definition of SE stands revised for more than 10 years. The result is the same as the finding of DeLorenzo et al., a significant number of patients experienced seizure lasting from 10 to 29 minutes [16]. They found seizure lasting 10–29 minutes represented over 35% of SE cases in their study period. During our study period, seizure episode lasting 10 to 29 minutes is 62.3% (38/61) of status epilepticus episode (Table 3). The difference could be related to the benefits of the national health insurance in Taiwan and to the fact that our patients could conveniently visit the hospital.

A previous study in the United States showed that the incidence of SE changed from 8.5% in 1991 to 4.9% in 1998 [3]. However, Dham et al. analyzed the discharge data of an American national hospital showing that the incidence of SE decreased from 13% in 1991 to 7% in 1999, but the incidence increased gradually from 7% in 1999 to 12.5% in 2010 [15]. The increasing incidence of SE is most likely attributed to more physicians following the ODSE.

In the episodes of seizures continuing for more than 5 min, 61.6% (61/99) of seizures persisted for more than 30 min. This recording is compatible with a previous study that reports that seizures without termination within 5 min may vary considerably in duration and last from many minutes to several hours [8]. Shinnar et al. showed that 24% of a first seizure will last longer than 30 min if it lasted longer than 5 min in patients aged younger than 19 years. In our study, 33.3% (4/12) of first seizure episodes lasted over 30 min in patients aged younger than 18 years when their seizures continued for over 5 min. Our study also found that when a patient's seizure continued for more than 5 min, around 40% of such seizures lasted between 5 and 30 min. The finding supports the view that the implementation of the ODSE in clinical practice is required and important.

In our study, 38.4% of the episodes lasted between 5 and 30 min and this may be related to early treatment in the emergency department. If we did not administer benzodiazepines or anticonvulsants to patients with a sustained seizure when they arrived at the emergency department, it is possible that more episodes would have persisted for longer than 30 min. In comparison to the patients classified as belonging to the TDSE group, significantly more patients aged younger than 18 years were classified as belonging to the UESE group (Table 2, *p* < 0.01). The first seizure lasting more than 5 min in patients aged younger than 18 years seems more likely to continue for 5–30 min; however, it persisted for over 30 min in patients aged older than 18 years and the patients with epilepsy at the same age (Table 4). Although the trend was not significant, this could change if the sample size was increased.

The study also found that the length of hospitalization and cost of treatment are not significantly different between the UESE group and patients not having SE. But they were lower in the UESE group than in the TDSE group. These results may be attributed to more than 50% of UESE patients being younger than 18 years and over one-third of the TDSE patients older than 60 years. Previous studies have shown that SE in older patients had poorer outcomes than those observed in younger patients [3]. Approximately 37.37% of episodes were underestimated in our hospital, indicating that only a small proportion of physicians followed the operational definition. SE defined by the operational definition was found to occur significantly more in patients aged younger than 18 years. In our study, we found that approximately 38% of SE cases were not diagnosed as SE because the physicians used TDSE. According to our results, previous studies reporting the incidence of SE may suffer from a significant rate of underestimation. Our results imply that when we want to investigate the trend regarding the incidence of SE, we must know previous studies may underestimate about one-third of SE.

These results highlight the importance of the continuing education of physicians and the importance of early diagnosis and treatment in patients with seizures.

However, our study has two limitations. (1) It is a hospital-based study, where most physicians do not use ODSE, and further large-scale investigations need to be conducted. (2) It is a retrospective study; the duration of seizure was obtained from the recording of chart. However, we have done our best to confirm the duration of seizure.

## Figures and Tables

**Table 1:** Characteristics of seizure (*N* = 256).

	No SE (*N* = 157)	TDSE (*n* = 61)	UESE (*n* = 38)	*p*
Gender				
Men	98 (62.4%)	43 (70.5%)	27 (71.1%)	
Women	59 (37.6%)	18 (29.5%)	11 (28.9%)	
Age				
<18	88 (56.1%)	18 (29.5%)	21 (55.2%)	
18–60	41 (26.1%)	21 (34.4%)	12 (31.6%)	
>60	28 (17.8%)	22 (36.1%)	5 (13.2%)	
Seizure type				
CTC	76 (48.4%)	23 (37.7%)	15 (39.5%)	
CPS	59 (37.6%)	29 (47.6%)	20 (52.6%)	
Partial seizure	15 (9.5%)	6 (9.8%)	3 (7.9%)	
Nonconvulsive		3 (4.9%)		
Other	7 (4.5%)			
Length of stay				
Median (days)	3	5	3	
Mean (days ± SD)	4.14 ± 3.64	9.49 ± 6.37	3.71 ± 2.52	<0.01^#^
Cost				
Median (NT)	16320	36218	13618	
Mean (NT ± SD)	24009 ± 30830	55049 ± 50501	18288 ± 20645	<0.01^#^

SE: status epilepticus, NT: Taiwan dollars, 1 USD = 30 NT, and ^#^one way ANNOVA.

**Table 2:** Characteristics of patients with status epilepticus.

	TDSE (*n* = 56)	UESE (*n* = 34)	*p*
Gender			
Men	38	23	1.0
Women	18	11	
Age			
<18	16	21	0.007
18–60	21	8	
>60	19	5	
Seizure type			
GTC	22	15	0.58
CPS	26	16	
Partial seizure	5	3	
Nonconvulsive	3	0	

GTC: generalized tonic clonic seizure, CPS: complex partial seizure.

**Table 3:** Duration of all seizure episodes.

Duration	Sex		Age (years)			Total
	F	M	<18	18–60	>60	
<5 minutes	59	98	88	41	28	157
5–9 minutes	0	8	1	6	1	8
10–29 minutes	12	18	20	6	4	30
≧30 minutes	18	43	18	21	22	61

**Table 4:** Duration of seizure in patients' first seizure and epilepsy.

	First seizure	Epilepsy	*p*
Age (0–18 yr)			
>30 min	4 (33.3%)	14 (51.9%)	0.284
5–30 min	8 (66.7%)	13 (48.2%)	
Age (>18 yr)			
>30 min	11 (61.1%)	32 (76.2%)	0.235
5–30 min	7 (38.9%)	10 (23.8%)	

**Table 5:** Causes of status epilepticus (*n* = 99).

	TDSE (*n* = 61)	UESE (*n* = 38)	Total	*p* value
Trauma	12	7	19	1.0
Mental retardation	9	6	15	1.0
Intracranial lesion	6	4	10	1.0
Stroke	12	6	18	0.8
Encephalitis	5	1	6	0.4
Metabolic factor	2	1	3	1.0
Alcohol	3	0	3	0.3
Cerebral palsy	2	1	3	1.0
Fever	1	2	3	0.5
Sepsis	1	0	1	1.0
Idiopathic	8	10	7	0.1
